# Estimating efficacy in trials with selective crossover

**DOI:** 10.1002/sim.7275

**Published:** 2017-03-15

**Authors:** Adam R. Brentnall, Peter Sasieni, Jack Cuzick

**Affiliations:** ^1^ Centre for Cancer Prevention, Wolfson Institute of Preventive MedicineQueen Mary University of LondonCharterhouse squareLondonEC1M 6BQU.K.

**Keywords:** binomial model, causal inference, compliance, proportional hazards model, switching

## Abstract

When one arm in a trial has a worse early endpoint such as recurrence, a data‐monitoring committee might recommend that all participants are offered the apparently superior treatment. The resultant crossover makes it difficult to measure differences between arms thereafter, including for longer‐term endpoints such as mortality and disease‐specific mortality. In this paper, we consider estimators of the efficacy of treatment on those who would not cross over if randomised to the apparently inferior arm. Binomial and proportional hazards maximum likelihood estimators are developed. The binomial estimator is applied to analysis of a breast cancer treatment trial and compared with intention‐to‐treat and inverse probability weighting estimators. Full and partial likelihood proportional‐hazard model estimators are assessed through computer simulations, where they had similar bias and variance. The new efficacy estimators extend those for all‐or‐none compliance to this important problem. © 2017 The Authors. *Statistics in Medicine* Published by John Wiley & Sons Ltd

## 1 Introduction

**Figure 1:**
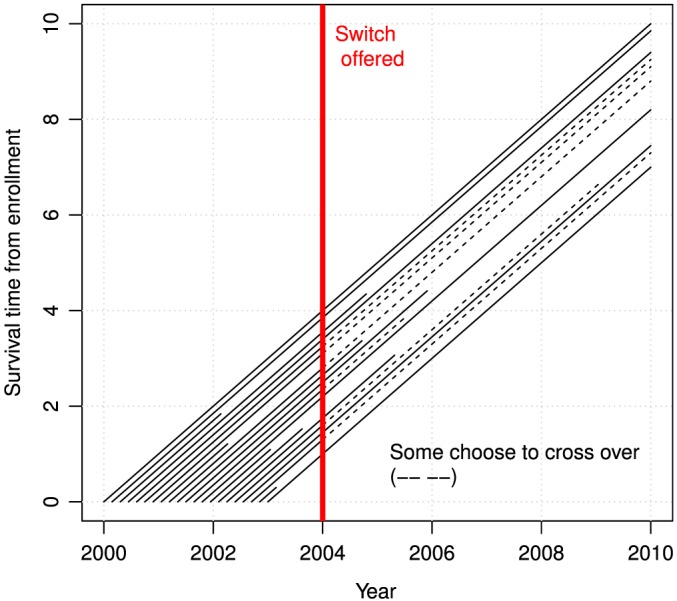
A lexis diagram representing survival and crossover. Each diagonal line is a person followed until they have the event and the line stops; censoring only occurs after 10‐year calendar time for clarity. [Colour figure can be viewed at wileyonlinelibrary.com]

Unplanned crossover occurs in randomised trials when participants decide to switch treatment arms. It makes it more difficult to measure a difference between arms, especially when there is selective crossover because those who switch have a different background risk than others. In this paper, we consider trials with selective crossover in one arm arising due to results from early efficacy endpoints, such as when initial results from a trial or other concurrent trials lead a data‐monitoring committee to recommend that all participants are offered the apparently superior treatment. This form of crossover is represented using a lexis diagram in Figure 1. It occurred in the BIG‐1 98 trial, where women were randomised to receive 5 years of either letrozole or tamoxifen to prevent breast cancer recurrence. Initial results 1 led the organisers to inform women in the tamoxifen monotherapy arms of their treatment, in order to allow an informed decision about future care; one quarter of those in the tamoxifen arm switched to letrozole 2.

The traditional inferential method for a trial with selective crossover is an intention‐to‐treat (ITT) analysis. This maintains the randomised balance of all causal factors other than the treatment, but it is likely to attenuate the estimated effect of treatment, because some participants in the control arm receive the intervention. A per‐protocol approach censors those who cross over in the analysis, but it is unreliable when crossover is informative because bias may occur in either direction and inference is compromised 3. Instead of these, we focus in this article on the effect of treatment on those who would not cross over when offered if randomised to control. Although this may also be biased for the effect of treatment on everyone in the trial, it is still of interest to identify the effect of treatment on those who would receive it for the duration, and these are the only individuals who provide information on treatment effects over the complete follow‐up period.

**Figure 2:**
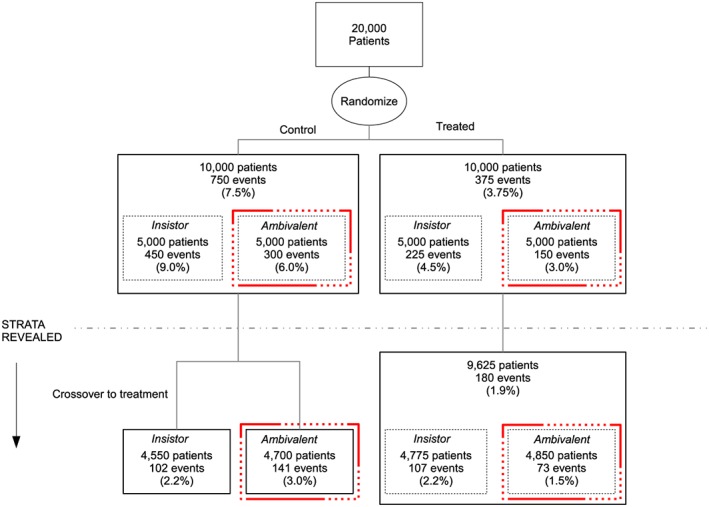
Hypothetical example to show the assumed latent strata in both trial arms when the endpoint is disease‐free survival. Each solid box contains observable data; efficacy compares ambivalents in the line‐dot boxes (— ‐ ‐ ‐). [Colour figure can be viewed at wileyonlinelibrary.com]

Efficacy is defined more formally using a model with two latent strata. The first latent stratum contains insistors, who would cross over to treatment when offered if randomised to control; the second consists of ‘ambivalents’, who would not cross over when offered if randomised to control. The strata are shown using an example in Figure 2. No one in the treatment arm switches to control, so there are assumed to be no individuals who defy randomisation by taking the opposite treatment, or who would always switch from treatment to control. The latent strata are unobserved in both arms prior to crossover, and only partially revealed in the control arm. Efficacy measures the average difference between the ambivalents in the two treatment arms.

**Figure 3:**
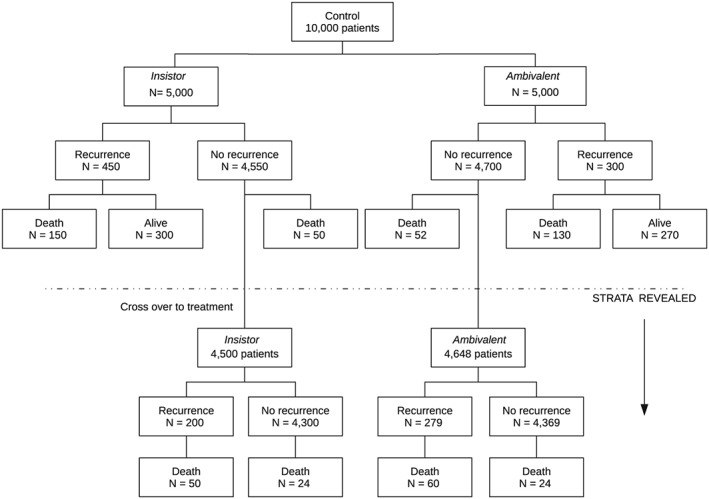
Hypothetical example to show assumed latent class structure in control when the endpoint is mortality.

Figure 2 is a suitable schematic for analysis of disease‐free survival such as in the BIG‐1 98 trial, but it does not adequately show subtle complications that arise for a mortality endpoint. Figure 3 illustrates the flow of patients under the model in the control arm when death is also included. The additional issue is that the underlying strata of participants in the control arm are not revealed after unblinding for those still alive but whose disease progressed beforehand. If this information was ignored, then the insistors in control with a recurrence before the offer to switch would be incorrectly identified as ambivalent.

We view the model as a plausible description of the trial population when there is a difference in underlying risk after crossover between those who switch treatment and the entire treatment arm. Models with similar latent strata have been applied to different problems 4, including when they are revealed at baseline 5, 6, 7 and when they are revealed after baseline 8. A consequence of the two‐strata model is that the proportion of insistors at risk is likely to be different at baseline from the time of potential crossover.

The unobserved boxes in Figure 2 show why the estimation problem is difficult. The marginal effect of treatment may be estimated before crossover by comparing the two arms without adjustment. After crossover, insistors in control receive the treatment. If the ambivalents were observed in both arms after the offer, then estimation of efficacy would be straightforward. The problem is that they are missing data before crossover in control and all the time in treatment. The remainder of the article considers this estimation problem.

## 2 Binomial model

A binary model may be applied to analysis of a trial when events are rare and little information is contained in the follow‐up or event time of patients. It also provides insight into assumptions and parameter identifiability aspects. We next focus on the model illustrated by Figure 2, but the methods may be adapted to mortality endpoints such as in Figure 3 by ensuring that the latent strata are unknown throughout follow‐up for those with a recurrence before the opportunity to cross over.

### 2.1 Assumptions

The first assumption is as follows:

A1 The potential time of crossover (time when the patient would cross over if an insistor and randomised to control) is observable for all participants in both arms.
  This holds for the type of crossover shown by Figure 1. Using A1, we may take two time periods before and after the offer (potential crossover time) and parameterise the probability *p*_*trk*_ of an event in period *t*=0,1 (before, after the offer), treatment arm *r*=0,1(control, treatment) and latent insistor stratum *k*=0,1 (ambivalent, insistor) as follows. For ambivalents (*k*=0) in control (*r*=0) and periods *t*=0,1
  $$p_{t00}=\alpha_{t},$$
  where $0⩽\alpha_{t}⩽1$ is an unknown probability of the event (if a constant rate $\alpha^{*}⩾0$ is assumed and the time at risk *T*_*t*_ is known, then *α*_*t*_=1−exp(−*α* ^∗^ *T*_*t*_), so that *α*_*t*_ in both periods only depends on a single unknown parameter). Efficacy *γ*_*t*_ in period *t*=0,1 is defined as the ratio between the treated and untreated ambivalents
  $$\gamma_{t}=p_{t10}/p_{t00}.$$
  Insistors in the treatment arm are taken to differ from the ambivalents in period *t*=0,1 through
  $$\omega_{t}=p_{t11}/p_{t10}.$$
  We also assume the following:
A2 The treatment effect prior to the offer is the same for the ambivalents and insistors, so that *p*_011_/*p*_001_=*γ*_0_, or equivalently
  $$p_{001}/p_{000}=\omega_{0}.$$
  A pragmatic reason for taking assumption A2 is that it ensures that the conditional treatment effect among ambivalents before the offer matches the marginal effect (seen hereafter in Equation (2), which would be the primary measure for an ITT analysis of a trial without selective crossover.
  After the offer to switch, we allow a different treatment effect for the insistors in control through
  $$p_{101}=\alpha_{1}\omega_{1}\gamma_{1}^{*},$$
  where $\gamma_{1}^{*}$ need not equal *γ*_1_. For example, a treatment crossover effect may exist, such as resulting from receiving a drug in control then the different drug from the treatment arm (which would not have been the case at baseline for those randomised to treatment). Alternatively, this might represent a difference in the treatment effect for insistors and ambivalents, and so testing whether $\gamma_{1}^{*}=\gamma_{1}$ is a way to partially verify A2, albeit after the offer. We require two further assumptions:
A3 The probability *π* of being in strata *k*=1(insistor) at baseline is the same in both arms because of randomisation.
A4 Censoring is independent of strata *k*=0,1.

A4 may be partly verified by using data in the control arm after the offer to switch and inspecting censoring in the other observed groups prior to and after the offer. Equivalent versions of A3 and A4 have been used by all‐or‐none compliance estimators 7.

### 2.2 Identifiability

Six observable statistics depend on the model parameters. These are the total number of events in arm *r*=0,1 in the first period *y*_0*r*_, the number of insistors *n*_101_ (or ambivalents *n*_100_) in control after the offer, the number of events in the second period in control ambivalents *y*_100_ and insistors *y*_101_, and the number of events in the treatment arm in the second period *y*_11_. The number censored *d*_0*r*_ in the first period in each arm *r* is an ancillary statistic, and binomial distributions are conditional upon the number *n*_*tr*_ in each arm *r* at risk at the start of each period *t*. The expected values of the observable statistics relate to the model as follows. Before the offer, the expected proportion of events in control is

(1) $$\text{E}(Y_{00})/n_{00}=\pi \alpha_{0}\omega_{0}+(1-\pi )\alpha_{0},$$

and in the treatment arm it is

(2) $$\text{E}(Y_{01})/n_{01}=\gamma_{0}E(Y_{00})/n_{00}.$$

At the offer to cross over, we observe the number of insistors still at risk in control *n*_101_, where

(3) $$\text{E}(N_{101})=\pi n_{00}-E(Y_{00})\left\{\frac{\pi \omega_{0}}{(1-\pi )+\pi \omega_{0}}\right\}-d_{00}\pi ,$$

which subtracts the expected number of events and censored (assumption A4) in insistors from the expected number enrolled in control at baseline. The term compounded by E(*Y*_00_) is the probability that the events are insistors. In the treatment arm, the number of insistors at risk at the offer *N*_111_ is unobserved, but the expected value has the same form as Equation (3) with *r*=1 in the subscripts rather than *r*=0. Thus, in the first period, there are three equations but four unknowns (*π*,*α*_0_,*ω*_0_,*γ*_0_). The only parameter that is uniquely identifiable without further constraints is the treatment effect *γ*_0_, replacing expectations by observed values in Equation (2). After the offer to switch the expected proportion of events in three observed groups (cf. Figure 2) is

(4) $$E(Y_{100})/n_{100}=\alpha_{1}$$

(5) $$E(Y_{101})/n_{101}=\gamma_{1}^{*}\alpha_{1}\omega_{1}$$

(6) $$E(Y_{11})/n_{11}=\gamma_{1}\{\pi_{11}\alpha_{1}\omega_{1}+(1-\pi_{11})\alpha_{1}\},$$

where *π*_11_ is the probability that individuals at risk in the treatment arm after the offer are insistors.

These equations help to assess identifiability of the model. Replacing the expectation with the observed value in Equation (4) provides an estimate of *α*_1_. There is one degree of freedom for ${\hat{\omega }}_{0}$ and $\hat{\pi }$(*π* may be expressed as a polynomial function of *ω*_0_ via Equation (3) and ${\hat{\pi }}_{11}=\hat{E}(N_{111})/n_{11}$). It might be important to allow for different rates in both periods, so that *α*_0_ and *α*_1_ cannot be tied together through an constant‐rate assumption. For example, risk of death increases with age. After incorporating (*α*_0_,*α*_1_,*ω*_0_,*γ*_0_), we have three unknowns $\left(\omega_{1},\gamma_{1},\gamma_{1}^{*}\right)$ and two degrees of freedom. However, this is not very restrictive because our a priori assumption is that $\gamma_{0}=\gamma_{1}=\gamma_{1}^{*}$, partly because we are interested in an overall measure of treatment efficacy over the entire period of follow‐up, and *ω*_0_=*ω*_1_ is also our primary model. The ability to check these assumptions contrasts favourably with an ITT analysis, where it would be very difficult to check whether the treatment effect is constant because any difference might only be caused by crossover.

### 2.3 Estimation

Estimation might be based on equating observed to expected in Equations (1)–(6), but we recommend to use maximum likelihood estimation, which will be covered in Section 4. For insight into the target of estimation, consider when $\gamma_{1}^{*}=\gamma_{1}$ and rearrange Equation (6) using Equations (4) and (5) so that

(7) $$\gamma_{1}=\frac{E(Y_{11})/n_{11}-\pi_{11}E(Y_{101})/n_{101}}{(1-\pi_{11})E(Y_{100})/n_{100}}.$$

The numerator is a total proportion of events in treatment after crossover minus the proportion of events in insistors after crossover, scaled by the proportion at risk at crossover in treatment. The denominator is the proportion of events in the ambivalents in control after crossover, scaled by the proportion of ambivalents at risk in treatment at crossover. This structure is very similar to for all‐or‐none compliance 6. Essentially, estimation of efficacy after crossover subtracts out the insistors from both arms by using the observed insistors from the control arm.

## 3 Proportional‐hazards model

The binomial model extends straightforwardly to an exponential model, but a proportional‐hazards model is more flexible, and so we focus on it next. Let the results from the trial for independent individuals *i*=1,…,*n* be (*t*_*i*_,*δ*_*i*_) where *t*_*i*_ is the continuous observation time and *δ*_*i*_ is equal to 1 if the event occurred and 0 if right censored at the last follow‐up. Denote treatment randomisation indicator *r*_*i*_ to be 1 if subject *i* was randomised to treatment and 0 if control, and *c*_*i*_=1 if an insistor and 0 if ambivalent. The potential crossover time is *s*_*i*_ (cf. Figure 1). Then the hazard at time t⩾0, conditional on covariates ***x***_*i*_=(*x*_*i*1_,…,*x*_*im*_) at baseline, is taken to be of form

(8) $$\lambda (t|r_{i},s_{i},c_{i},x_{i};\theta )=\lambda_{0i}(t)\psi (t,r_{i},s_{i},c_{i},x_{i};\theta ),$$

where *λ*_0*i*_(*t*) is a baseline hazard function (with an *i* subscript because it might be stratified so that *λ*_0*i*_=*λ*_0*j*_ if *i* and *j* are in the same strata, or not with *λ*_0*i*_=*λ*_0_), and

(9) $$\psi (t,r_{i},s_{i},c_{i},x_{i};\theta )=\exp[\gamma \{r_{i}+I(t⩾s_{i})(1-r_{i})c_{i}\}+\omega c_{i}+x_{i}\beta^{\prime }],$$

where *I*(.) is the indicator function, prime denoting the transpose, and parameters ***θ***=(*γ*,*ω*,*β*_1_,…,*β*_*m*_). The treatment effect parameter is *γ*, the effect of being an insistor *ω*, and covariate effects ***β***=(*β*_1_,…,*β*_*m*_). The formulation is an extension of 7, but where the insistors receive the effect of treatment only after crossing over. It is quite different than treating compliance as a time‐dependent covariate, which does not respect randomisation and leads to bias, because it allows for the latent strata in both arms. The model does not include time‐varying covariates, but this is unlikely to be restrictive for randomised trials, where even static covariates are not commonly used other than by stratifying the baseline hazard function.

The estimators that we develop in the next section require assumptions A1–A3 from Section 2 and the following.

A4b Censoring is independent given covariates and independent of latent strata given covariates.
A5a Latent strata are independent of covariates, or
A5b latent strata are independent of covariates within defined groups.
A6 Latent strata are independent of time at entry within defined groups.

A5a is needed for the partial likelihood estimator in the Section 4; the more relaxed A5b is for the full likelihood estimator. Both are not an issue if no covariates are used in the model, and A5a was also used by 7 in their partial‐likelihood estimator. A6 is needed for both estimators. The assumptions A5 and A6 may be checked using the data after the offer to switch. If A5b and A6 are not met without groups, then groups might be sought where they are met. As for the binomial estimator, one may test if the treatment effect is constant before and after the offer.

An outline of a full maximum likelihood estimation algorithm is as follows; see Section 4 for technical details. Firstly, the probability each individual is an insistor at baseline is computed given $\hat{\theta }$; then the baseline hazard may be fitted conditional upon this and $\hat{\theta }$; finally, ***θ*** are fitted given the baseline hazard and insistor probability estimates. The approach used in this algorithm to estimate the proportion of insistors at baseline (full likelihood) and through time (partial likelihood) is the biggest difference compared with 7. This is because in the all‐or‐none compliance case the proportion of switchers at risk through time in the unobserved arm may be estimated simply as the observed number of switchers at risk. However, the new algorithm could also be applied in the all‐or‐none case where the main practical difference is that the estimate of the number of non‐compliers at risk in the unobserved arm would change each time there is an event in that arm, rather than only each time there is an event in the observed arm. The overall profile‐likelihood algorithm continues until convergence in the likelihood 9. The partial likelihood is asymptotically concave because it is the same form as for all‐or‐none compliance 7, so it offers a good starting point for full maximum likelihood.

Inference may be based on profile likelihood. One approach that is not recommended is to use Wald confidence intervals from the partial likelihood; these would underestimate variability by treating the proportion of insistors at risk through time as known.

## 4 Maximum likelihood estimation

### 4.1 Binary model

In the first period in control (*t*=0;*r*=0) and the treatment arm in both periods (*t*=0,1;*r*=1), the probability of an event is

(10) $$p_{tr}=\pi_{tr}p_{tr1}+(1-\pi_{tr})p_{tr0},$$

where *p*_*trk*_ was defined in Section 2 and *π*_0*r*_=*π* is assumed constant for *t*=0. There are five unknown parameters in the primary model: the baseline probabilities *α*_0_,*α*_1_(potentially different in both periods), the insistor effect *ω*=*ω*_0_=*ω*_1_ (common in both periods), the treatment effect $\gamma =\gamma_{0}=\gamma_{1}=\gamma_{1}^{*}$(common in both periods) and the proportion of insistors at baseline *π*. The probability of being an insistor at risk in treatment after the offer (*π*_11_) is estimated as a function of the data and parameter estimates (*α*_0_,*ω*,*γ*,*π*), as discussed in Section 2. For the treatment arm (*t*=0,1;*r*=1) and the control arm before the offer (*t*=0;*r*=0), the likelihood is

(11) $$L_{tr}\propto p_{tr}^{y_{tr}}{\left(1-p_{tr}\right)}^{n_{tr}-y_{tr}}$$

and after the offer in control

(12) $$L_{10}\propto \overset{1}{\underset{k=0}{\prod }}p_{10k}^{y_{10k}}{\left(1-p_{10k}\right)}^{n_{10k}-y_{10k}}.$$

The complete likelihood

(13) $$L\propto \overset{1}{\underset{t=0}{\prod }}\overset{1}{\underset{r=0}{\prod }}L_{rt}$$

may be used to form maximum likelihood estimates and profile‐likelihood confidence intervals in the usual manner.

### 4.2 Proportional‐hazards model

#### 4.2.1 Likelihood function

Before crossover and in the treatment group, we do not observe the latent strata. Thus, if *t*_*i*_<*s*_*i*_ and *r*_*i*_=0, or when *r*_*i*_=1, then the likelihood for individual *i* is

(14) $$L_{i}(\theta |r_{i},s_{i},x_{i})=\pi_{i}L_{i}(\theta |r_{i},c_{i}=1,x_{i})+(1-\pi_{i})L_{i}(\theta |r_{i},c_{i}=0,x_{i})$$

where *π*_*i*_=*P*(*C*_*i*_=1|*r*_*i*_,***x***_*i*_) is the probability a person is an insistor conditional on baseline covariates ***x***_*i*_ and

$$L_{i}(\theta |r_{i},c_{i},x_{i})=\lambda {\left(t_{i}|r_{i},c_{i},x_{i};\theta \right)}^{\delta_{i}}\exp\left\{-\int_{0}^{t_{i}}\lambda (u|r_{i},c_{i},x_{i};\theta )du\right\}.$$

If the crossover is observed ( $t_{i}⩾s_{i}$ and *r*_*i*_=0), then *L*_*i*_ is the product of two terms given later in Equation (20). The first contribution is a survivor function *P*(*t*_*i*_>*s*_*i*_|*r*_*i*_=0,*s*_*i*_,***x***_*i*_), which is of the same form as Equation (14); the second contribution when *c*_*i*_ is known ( $t_{i}⩾s_{i}$) is $\lambda {\left(t_{i}|r_{i},s_{i},c_{i},x_{i};\theta \right)}^{\delta_{i}}\exp\left\{-\int_{s_{i}}^{t_{i}}\lambda (u|r_{i},c_{i},x_{i};\theta )du\right\}$. The full likelihood for everyone is

$$L(\theta )=\overset{n}{\underset{i=1}{\prod }}L_{i}(\theta |r_{i},s_{i},x_{i}),$$

and we will write *l*(***θ***), or just *l*, for the log likelihood.

#### 4.2.2 Partial likelihood function

Consider when *λ*_0*i*_(*t*)=*λ*_0_(*t*) so that there are no baseline‐hazard groups. Then the partial likelihood is denoted

(15) $$PL(\theta )=\overset{n}{\underset{i=1}{\prod }}{\left\{\frac{\eta (t_{i},r_{i},s_{i},x_{i})}{\overset{n}{\underset{j=1}{\sum }}I(t_{j}⩾t_{i})\eta (t_{i},r_{j},s_{i},x_{j})}\right\}}^{\delta_{i}}.$$

When the latent stratum is unknown (*r*_*j*_=0 and *s*_*j*_>*t*_*i*_, or *r*_*j*_=1)

$$\eta (t_{i},r_{j},s_{j},x_{j})=\pi (t_{i}|r_{j})\psi (t_{i},r_{j},s_{j},c_{j}=1,x_{j})+\{1-\pi (t_{i}|r_{j})\}\psi (t_{i},r_{j},s_{j},c_{j}=0,x_{j}),$$

where *π*(*t*_*i*_|*r*_*j*_) is the conditional probability of being an insistor (*c*_*j*_=1) still at risk in arm *r*_*j*_ at time *t*_*i*_. Note that we follow assumption A5a and assume *π*(*t*_*i*_|*r*_*j*_,***x***_*j*_)=*π*(*t*_*i*_|*r*_*j*_). When crossover status is known (*r*_*j*_=0 and $s_{j}⩽t_{i}$), then *η*(*t*_*i*_,*r*_*j*_,*s*_*j*_,***x***_*j*_)=*ψ*(*t*_*i*_,*r*_*j*_,*c*_*j*_,***x***_*j*_). For a model with different baseline hazard functions for defined groups, the product of different partial likelihoods of form (15) for each baseline‐hazard group is taken.

#### 4.2.3 Proportion of insistors

In this section, we develop a consistent estimator of the proportion of insistors at risk through time given the event times, insistor effect *ω* and the number of insistors at baseline *u*, without a full probability survival model.

We take a group where *C* is independent of ***X*** (assumption A5b) and time at entry in the group (A6); the following may be applied separately to all such groups. Then consider arm 0 where latent strata are observed for $t_{j}⩾s_{j}$. The proportion of insistors at risk is to be updated at $t_{j}^{\prime }=\min(t_{j},s_{j})$ for *j*=1,…,*n*_0_ individuals in arm 0, which is the crossover time *s*_*j*_ if it is before the survival time *t*_*j*_, or *t*_*j*_ otherwise. The combination of censoring indicator *δ*_*j*_ and whether the individual was at risk at *s*_*j*_ is classified using

$$e_{j}=\left\{\begin{array}{ccc} 0 & s_{j}>t_{j}\;\text{and}\;\delta_{j}=1 & \text{(event before offer to switch)}\\ 1+c_{j} & s_{j}⩽t_{j} & \text{(at risk after offer)}\\ 3 & s_{j}>t_{j}\;\text{and}\;\delta_{j}=0 & \text{(censored before the offer)}. \end{array}\right.$$

We take the order of individuals to be sorted ascending by $t_{j}^{\prime }$, so that for indices $j\in (1,\ldots ,n_{0}-1),t_{j}^{\prime }⩽t_{j+1}^{\prime }$. For a given *u*_0_=*u* number of insistors in arm 0 at baseline and insistor effect $\omega^{\prime }=\exp(\omega )$, the number *u*_*j*_ and proportion of insistors at risk for *j*=1,…,*n*_0_ are estimated iteratively using

(16) $$\xi_{j}=\left\{\begin{array}{ccc} \hat{\pi }(t_{j-1}^{\prime })\omega^{\prime }/\{(1-\hat{\pi }(t_{j-1}^{\prime }))+\hat{\pi }(t_{j-1}^{\prime })\omega^{\prime }\} & e_{j}=0 & \text{(unknown stratum, event before the offer)}\\ 0 & e_{j}=1 & \text{(ambivalent)}\\ 1 & e_{j}=2 & \text{(insistor)}\\ \pi (t_{j-1}^{\prime }) & e_{j}=3 & \text{(unknown stratum, censored before the offer)} \end{array}\right.$$

so that $\hat{\pi }(t_{j}^{\prime })={û}_{j}/(n_{0}-j)$ where ${û}_{j}={û}_{j-1}-\xi_{j}$. The rationale is as follows. When *e*_*j*_=1,2, then we know whether the individual crossed over, and the number still at risk ${û}_{j}$ at time $t_{j}^{\prime }$ is updated accordingly. When *e*_*j*_=3, then $\hat{\pi }(t_{j}^{\prime })$ does not change because censoring is assumed independent of insistor status. If *e*_*j*_=0, then *c*_*j*_ is unobserved and *ξ*_*j*_ estimates the probability *P*(*c*_*j*_=1|*e*_*j*_); compare with Equation (3) for the binomial model. It arises from model (8) where

(17) $$E\{dN(t_{j}^{\prime }|c_{j}=1)\}=\frac{\pi (t_{j}^{\prime })\omega^{\prime }}{1-\pi (t_{j}^{\prime })}E\{dN(t_{j}^{\prime }|c_{j}=0)\},$$

using *N*(*t*|*c*_*j*_) to denote a counting process that jumps by one when there is an event. For example, if the arms are balanced with $\pi (t_{j}^{\prime })=0.5$, but the insistor effect $\omega^{\prime }=2$, then for each event among the ambivalents, (*c*_*j*_=0) $\omega^{\prime }=2$ events are expected in the insistors (*c*_*j*_=1), that is, $E\{dN(t_{j}^{\prime }|c_{j}=1)\}=2\times E\{dN(t_{j}^{\prime }|c_{j}=0)\}$. Similarly, if $\omega^{\prime }=1$ and there are twice as many insistors as ambivalents ( $\pi (t_{j}^{\prime })=2/3$), then for each event among ambivalents, there will be $\pi (t_{j}^{\prime }){\left\{1-\pi (t_{j}^{\prime })\right\}}^{-1}=2$ events expected in the insistors. Although the survival model is in continuous time, a few ties are sometimes tolerated. If $t_{j}^{\prime }=t_{j+1}^{\prime }=\ldots =t_{j+k}^{\prime }$ are tied, then the suitable adjustment is to set the associated ${û}_{l}={û}_{j+k}$ for *l*=*j*,…,*j*+*k*−1.

Secondly, the number of insistors at baseline *u* may be estimated by maximum likelihood, given *ω*. The data are occasions when an individual crossed over or not, that is, when *e*_*j*_=1,2. For *j*=1,…,*n*_0_, if *e*_*j*_=0 or 3, then the log‐likelihood *l*_*j*_(*u*|*ω*)=0, else

(18) $$l_{j}(u|\omega )=(e_{j}-1)\log\{\pi^{*}(t_{j}^{\prime })\}+(2-e_{j})\log\{1-\pi^{*}(t_{j}^{\prime })\},$$

where the $\pi^{*}(t_{j}^{\prime })=\pi (t_{j}^{\prime })$ if $t_{j}^{\prime }>t_{j-1}^{\prime }$, otherwise $\pi^{*}(t_{j}^{\prime })=\pi (t_{j-k}^{\prime })$ where *k* selects the first crossover event time equal to $t_{j}^{\prime }$. The $\pi^{*}(t_{j}^{\prime })$ definition is needed for ties when more than one event occurs at each point $t_{j}^{\prime }$; the same estimate of the proportion of insistors should be used for all of them. The proportion of insistors at the start may be estimated by maximising the overall log‐likelihood $l(u|\omega )=\sum_{j=1}^{n_{0}}l_{j}(u|\omega )$.

Finally, given *u* and *ω*, one may estimate the proportion of insistors at risk in the observed arm 0. If the proportion of insistors at the start of the trial in arm 0 is estimated to be the same as in arm 1, then one may use Equation (16) but for the unobserved arm 1.

#### 4.2.4 Baseline hazard function

A baseline hazard function may be estimated for each baseline‐hazard group, so for simplicity take that *λ*_*i*0_(*t*)=*λ*_0_(*t*) for *i*=1,…,*n*, where *λ*_0_(*t*) is a step function with unknown jumps Δ_*i*_ at each observed event time *t*_*i*_ and that the times are sorted ascending. To estimate it, we use the partial derivativ

$$\frac{\partial l}{\partial \Delta_{i}}=\overset{n}{\underset{j=1}{\sum }}\frac{\partial l_{j}}{\partial \Delta_{i}}.$$

In the following, we drop conditioning arguments from the setup except *c* in reduce notational burden, but they are still present. Firstly, consider *j*=1,…,*n* where *r*_*j*_=1, that is, those in the treatment arm whose crossover status *c*_*j*_ is not observed. Let *π*_*j*_=*P*(*C*_*j*_=1|***x***_*j*_), then in this case

$$L_{j}=\pi_{j}\lambda_{j}^{\delta_{j}}(c_{j}=1)\exp\{-\Lambda (c_{j}=1)\}+(1-\pi_{j})\lambda_{j}^{\delta_{j}}(c_{j}=0)\exp\{-\Lambda (c_{j}=0)\}$$

so that

$$l_{j}=\log(\Delta_{j}^{\delta_{j}})+\log(A_{j})$$

where

$$A_{j}=\pi_{j}\psi {\left(c_{j}=1\right)}^{\delta_{j}}\exp\{-\Lambda (c_{j}=1)\}+(1-\pi_{j})\psi {\left(c_{j}=0\right)}^{\delta_{j}}\exp\{-\Lambda (c_{j}=0)\}.$$

For *j*=*i*,…,*n*, because

$$\partial \exp\{-\Lambda (t_{j})\}/\partial \Delta_{i}=-\psi \exp\{-\Lambda (t_{j})\},$$

we have that, for *j*=*i*,…,*n* such that *r*_*j*_=1,

(19) $$\begin{array}{ccc} \frac{\partial l_{j}}{\partial \Delta_{i}} & =\delta_{i}/\Delta_{i}-\overset{n}{\underset{j=i}{\sum }}[\pi_{j}\psi {\left(c_{j}=1\right)}^{\left(1+\delta_{j}\right)}\exp\{-\Lambda (t_{j}|c_{j}=1)\} & \\ & \;+(1-\pi_{j})\psi {\left(c_{j}=0\right)}^{\left(1+\delta_{j}\right)}\exp\{-\Lambda (t_{j}|c_{j}=0)\}]/A_{j}\\ & :=\delta_{i}/\Delta_{i}-\overset{n}{\underset{j=i}{\sum }}a_{1j}\\ & :=\delta_{i}/\Delta_{i}+a_{1}, \end{array}$$

where := denotes definition. Now consider *j*=1,…,*n* in arm 0. When insistor status is not observed because *t*_*j*_<*s*_*j*_, then the same form as Equation (19) applies; denote it *a*_2_. If $t_{j}⩾s_{j}$, then

(20) $$\begin{array}{ccc} L_{j} & =[\pi_{j}\exp\{-\Lambda (s_{j}|c_{j}=1)\}+(1-\pi_{j})\exp\{-\Lambda (s_{j}|c_{j}=0)\}] & \\ & \;\times \lambda {\left(t_{j}|c_{j}\right)}^{\delta_{j}}\exp\{-\Lambda (s_{j},t_{j}|c_{j})\} \end{array}$$

where $\Lambda (s_{j},t_{j}|c_{j})=\int_{s_{j}}^{t_{j}}\lambda_{j}(v|c_{j})dv$, so that

$$l_{j}=\log\{A_{j}^{*}\}+\log(\Delta_{j}^{\delta_{j}})-\Lambda (s_{j},t_{j}|c_{j})+\log(\psi^{\delta_{j}})$$

where

$$A_{j}^{*}=\pi_{j}\exp\{-\Lambda (s_{j}|c_{j}=1)\}+(1-\pi_{j})\exp\{-\Lambda (s_{j}|c_{j}=0)\}.$$

Then for *j*=*i*,…,*n* such that $s_{j}⩽t_{i}$

$$\begin{array}{ccc} \frac{\partial l_{j}}{\partial \Delta_{i}} & =-\psi (c_{j})+I(j=i)\delta_{j}/\Delta_{i} & \\ & :=a_{3j}^{\left(1\right)} \end{array}$$

and for when *s*_*j*_>*t*_*i*_

$$\begin{array}{ccc} \frac{\partial l_{j}}{\partial \Delta_{i}} & =-[\pi_{j}\psi (c_{j}=1)\exp\{-\Lambda (s_{j}|c_{j}=1)\}+ & \\ & \;(1-\pi_{j})\psi (c_{j}=0)\exp\{-\Lambda (s_{j}|c_{j}=0)\}]/A_{j}^{*}+I(j=i)\delta_{j}/\Delta_{i}\\ & :=a_{3j}^{\left(2\right)}, \end{array}$$

so that overall for those observed to crossover (*t*_*j*_>*s*_*j*_)

$$\begin{array}{ccc} \frac{\partial l}{\partial \Delta_{i}} & =\overset{n}{\underset{j=i}{\sum }}a_{3j}^{\left(1\right)}I(s_{j}<t_{i})+a_{3j}^{\left(2\right)}I(s_{j}⩾t_{i}) & \\ & :=a_{3}. \end{array}$$

Overall, we have

(21) $$\frac{\partial l}{\partial \Delta_{i}}=I(r_{i}=1)a_{1}+I(r_{i}=0)\{I(t_{i}<s_{i})a_{2}+I(t_{i}⩾s_{i})a_{3}\}.$$

Setting Equation (21) to zero provides a route to estimation of Δ_*i*_ for *i*=1,…,*n*, conditional on ***θ*** and *π*. If *δ*_*i*_=1 and *δ*_*i*+1_=1 and it is not true that *t*_*i*_<*s*_*j*_<*t*_*i*+1_ for *j*=*i*+1,…,*n*, then

(22) $$\begin{array}{ccc} \Delta_{i}^{-1}-\Delta_{i+1}^{-1} & =I(r_{i}=1)a_{1i}+I(r_{i}=0)\{I(t_{i}<s_{i})a_{2i}+I(t_{i}⩾s_{i})a_{3i}\} & \\ & :=D_{i}^{*} \end{array}$$

say. This simple form arises because the *i*+1,…,*n* terms in the *a*_1_,*a*_2_,*a*_3_ summations over *j* cancel out. Extra terms are needed with censoring, and if *t*_*i*_<*s*_*j*_<*t*_*i*+1_ for *j*=*i*+1,…,*n* because the derivatives of those *j*'s likelihood contribution with respect to Δ_*i*_ do not cancel out. Thus, more generally for two events at times *i* and *i*+*k* with censored observations between from *i*+1,…,*i*+*k*−1

(23) $$\begin{array}{ccc} \Delta_{i}^{-1}-\Delta_{i+k}^{-1} & =\overset{i+k-1}{\underset{j=i}{\sum }}D_{j}^{*}+\overset{n}{\underset{j=i+k}{\sum }}I(r_{j}=0)I(t_{i}<s_{j}⩽t_{i+k})(a_{3j}^{\left(2\right)}-a_{3j}^{\left(1\right)}) & \\ & :=D_{i} \end{array}$$

say, where the first summation is for the censored observations and the second summation is for those observed to crossover in arm 0 between *t*_*i*_ and *t*_*i*+*k*_. Now $\Delta_{i+1}^{-1}=\Delta_{i}^{-1}-D_{i}$, and the problem of estimating the baseline given ***θ*** is reduced to determining Δ_*i*_ for the first *i*(i.e. smallest *t*_*i*_) with *δ*_*i*_=1. This may be achieved using a root‐finding algorithm for the score function.

The above extends when there are ties. Suppose that *w*_*i*_ times are tied following *t*_*i*_(*t*_*i*_=*t*_*i*+1_,…,*t*_*i*+*w*−1_), and *w*_*i*+*k*_ following *t*_*i*+*k*_. Then the left‐hand side of Equation (23) is $w_{i}\Delta_{i}^{-1}-w_{i+k}\Delta_{i+k}^{-1}$.

## 5 Example

We next apply the binomial estimator to the data from two monotherapy arms in the BIG1‐98 trial, where postmenopausal women who had been diagnosed with hormone‐receptor positive invasive breast cancer were randomised to receive 5 years of tamoxifen (control) or letrozole (treatment). Following the primary analysis 1, women who were still receiving tamoxifen were unblinded 2. In this example, one might question the model assumption that the treatment effect of letrozole is that same after taking tamoxifen, as at baseline (i.e. $\gamma_{1}=\gamma_{1}^{*}$ in the notation of Section 2). This issue was actually investigated by planned crossover arms in the BIG‐1 98 trial, where little difference was found 10, so we proceed under the assumption.

Table 1 shows a summary of data previously reported 10, 11. The results before unblinding are in period 0; period 1 is the time after that until 12‐year follow‐up. Of 2459 women randomised to tamoxifen, some 25% received letrozole after unblinding, with most crossing over between 3 and 5 years since randomisation.

**Table 1:** Disease‐free survival results from BIG‐1 98 trial 10, 11.

	Period	At risk	Number (%) events
Letrozole	0	2463	352 (14.3)
Tamoxifen	0	2459	418 (17.0)
Letrozole	1	2045	294 (13.0)
Tamoxifen	1	1975	309 (13.8)
– do not cross over	1	1356	251 (15.5)
– cross over	1	619	58 ( 9.4)

The data in period 0 were reported by 11. The number of events in period 1 are the 12 year follow‐up analysis 10 minus events in period 0. The number at risk in the second period was calculated as the number randomised minus the number (i) of disease‐free survival events in the first period, (ii) lost to follow up and (iii) withdrawn (Table A2 in 11), split by the reported 619 who chose to cross over 10.

Table 2 shows published results from fitting ITT and inverse probability of censoring weighting (IPCW) stratified proportional hazards models using individual time‐to‐event data 10. The per‐protocol approach that censors women who ceased to comply with their randomised allocation is not suitable for these data because there was strong evidence that those who crossed over had a different baseline risk than those who did not; the group who crossed over from tamoxifen in Table 1 had a lower proportion of events (9.4%) than the letrozole arm (13.0%). Thus, the randomised balance of risk factors between comparison groups is almost certainly lost in a per‐protocol analysis.

**Table 2:** Disease‐free survival treatment effect estimates (95% CI) from BIG‐1 98 trial 12‐year update 10.

		Estimate	(95%CI)
Hazard ratio	ITT	0.86	(0.78, 0.96)
	IPCW	0.82	(0.74, 0.92)
Relative risk	ITT	0.89	(0.81, 0.97)
	Efficacy ( $\hat{\gamma })$	0.86	(0.77, 0.96)
	– *t*=0 ( ${\hat{\gamma }}_{0}$)	0.84	(0.74, 0.96)
	– *t*=1 ( ${\hat{\gamma }}_{1}$)	0.90	(0.74, 1.07)
	– [Het. test]		$\left[\chi_{1}^{2}=0.32\right]$

Het. test, heterogeneity likelihood‐ratio test.

An ITT analysis was applied using just the number of events and women in both arms. In general, relative risks are closer to unity than hazard ratios, so it is not surprising that the relative risks for ITT were closer to unity than the hazard ratio in Table 2. However, differences were small and ITT inference was unchanged. The binomial efficacy estimate for disease‐free survival was of a similar order less than the ITT estimate, in comparison with the IPCW estimate. This provides some support to the IPCW estimate for disease‐free survival.

**Figure 4:**
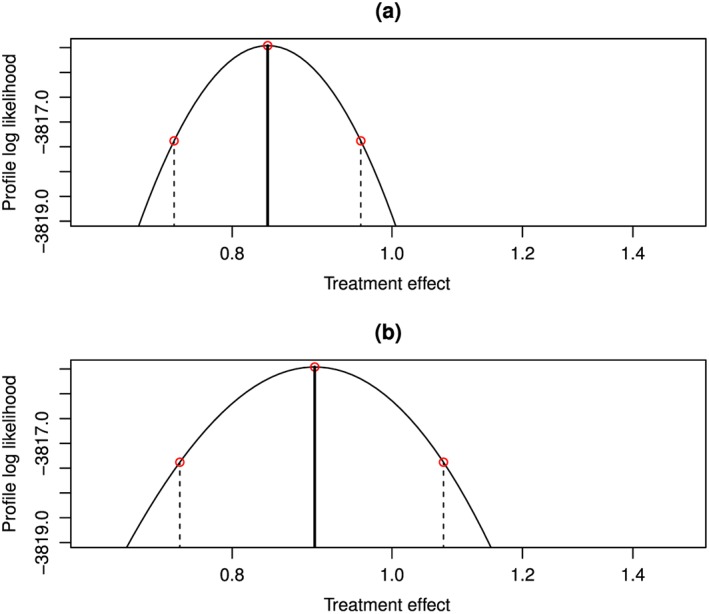
Profile likelihood surface to obtain confidence intervals for separate treatment effects in (a) the first period and (b) the second period, for disease‐free survival in the example. The dotted lines and points are the 95% confidence interval. [Colour figure can be viewed at wileyonlinelibrary.com]

We also used the model to allow for different treatment effects in each period. There was little heterogeneity between the point estimates of efficacy before and after unblinding (Table 2; Figure 4). However, the wide confidence intervals after crossover indicate that further follow‐up would be useful to determine whether disease‐free survival was better in the first period. There was also almost no evidence for a difference in *ω*_0_ and *ω*_1_ (data not shown).

A limitation of the example is that it is best viewed as a demonstration of the method. Firstly, the actual number at risk in the second period was only estimated on the basis of published data (although small changes to these numbers will not materially affect results). Secondly, application of the proportional‐hazards model on individual data would also be a better comparison with IPCW. Finally, we were unable to apply either estimator for mortality endpoints because the subdivision of data illustrated by Figure 3 would be needed.

## 6 Simulations

The following simulations consider the performance of the full and partial likelihood estimators for different treatment and insistor effects, as the level of information on the treatment effect changes from censoring. Nelder–Mead simplex algorithms were used for both full and partial likelihood estimation, but very little difference was seen to a Broyden–Fletcher–Goldfarb–Shanno algorithm (not reported); Brent–Dekker algorithms estimated the insistor proportions and baseline hazard function 12.

Survival times were simulated from an exponential distribution. The simulation was set up so that there were three periods in follow‐up time: before, during and after crossover. The period during crossover was the same length as the period before it. We considered two censoring scenarios (i) with censoring where an expected 5% events occurred before the first crossover in the lowest hazard group, and 5% events after the last crossover, and (ii) no censoring with an expected 10% of events before the first crossover in the lowest hazard group. For example, if the hazard was less for insistors and the treatment group, then the lowest hazard group was insistors randomised to treatment. One thousand individuals were in each arm, and the chance of being an insistor was 25% in both arms, but the number at baseline was sampled independently in both arms from a binomial distribution. The treatment effect (*γ*) was 7/10 or 10/7; the effect of being an insistor (*ω*) was either 0.2 or 2.0.

The pseudo algorithm used to generate the data is as follows. For *r*=0,1 and *i*=1,…,*n*_*r*_ individuals in arm *r* with maximum follow‐up *F* and an event‐rate pre‐crossover *λ*_*ar*_(*c*_*i*_) and post‐crossover *λ*_*pr*_(*c*_*i*_) conditional on underlying crossover trait *c*_*i*_: sample (i) crossover trait *c*_*i*_ at baseline from *P*(*c*_*i*_=1)=0.25; (ii) crossover time *s*_*i*_ from a uniform distribution between 0 and 1 {*U*(0,1)} plus 1; (iii) survival time *t*_*i*1_ from an exponential distribution with pre‐crossover rate *λ*_*ar*_(*c*_*i*_); (iv) survival time *t*_*i*2_ from an exponential distribution with post‐crossover rate *λ*_*pr*_(*c*_*i*_). Then calculate (i) unobserved survival time (no censoring) $t_{i}^{u}=t_{i1}$ if $t_{i1}⩽s_{i}$; else $t_{i}^{u}=s_{i}+t_{i2}$; (ii) observed survival time (with censoring) *t*_*i*_= min( $t_{i}^{u},F$); and (iii) event status *d*_*i*_=1 if *t*_*i*_<*F*; else *d*_*i*_=0.

Full and partial likelihood estimators are compared in Table 3. Mean bias was very small; for example, 0.467 for $\hat{\gamma }$ from the partial likelihood in scenario 4 implies that the mean estimated treatment effect was 0.699=exp{(1+0.00467)×log(0.7)}. The full likelihood bias was less than the partial likelihood for both the treatment effect *γ* and insistor effect *ω*, but their variances were very similar. There was also very little difference in the estimated proportion of insistors at baseline. Thus, overall, the full and partial likelihood estimators had similar performance in a setting where the model assumptions were met, and assumption A5a was appropriate.

**Table 3:** Simulation results of FL and PL estimators.

Scenario	1	2	3	4	5	6	7	8
Treatment exp(*γ*)	7/10	7/10	7/10	7/10	10/7	10/7	10/7	10/7
Switching exp(*ω*)	0.2	0.2	2.0	2.0	0.2	0.2	2.0	2.0
Censoring	Y	N	Y	N	Y	N	Y	N
(a) Mean bias (%) of log estimates								
*γ* FL	−0.002	0.004	−0.004	0.003	0.006	−0.003	0.007	0.001
*γ* PL	−0.231	0.340	0.533	0.467	0.666	−0.654	−0.037	−0.275
*ω* FL	0.009	−0.003	−0.019	0.002	0.015	0.002	−0.003	0.001
*ω* PL	0.835	−0.527	−1.078	0.130	1.522	−0.069	−0.259	−0.033
(b) Variance of log estimates								
*γ* FL	0.485	0.377	1.388	0.289	0.515	0.449	1.281	0.274
*γ* PL	0.485	0.407	1.405	0.289	0.519	0.484	1.284	0.282
*ω* FL	6.597	2.295	5.944	0.994	5.067	1.194	3.831	1.409
*ω* PL	6.612	2.066	6.005	0.993	5.078	1.210	3.849	1.399
(c) Percent (%) of insistors at baseline estimates $\hat{\pi }$
Mean FL	25.056	25.116	24.971	25.042	24.998	25.073	25.016	25.034
Mean PL	25.059	25.118	24.989	25.041	24.997	25.091	25.020	25.022
SD FL	1.579	1.910	1.732	1.848	1.447	1.589	1.786	2.013
SD PL	1.580	1.741	1.736	1.848	1.447	1.603	1.787	2.025

FL, full likelihood; PL, partial likelihood.

To evaluate the use of a simple bootstrap for inference, Table 4 presents some results of resampling with replacement individuals in each arm in each simulation sample. Two hundred bootstrap resamples were made in each sample, and an estimate of the variance of the parameter estimate was obtained. The table shows that this matched the observed variance from the 1000 simulation replicates at each scenario quite closely. Because the variance of the full likelihood matched that of the partial likelihood quite closely in Table 3, one might just bootstrap the partial likelihood as computational time becomes more of a consideration.

**Table 4:** Bootstrap estimates of parameter uncertainty using partial likelihood estimator.

Scenario	1	2	3	4	5	6	7	8
Var( $\hat{\gamma }$)	0.485	0.407	1.405	0.289	0.519	0.484	1.284	0.282
Mean bootstrap	0.503	0.412	1.450	0.295	0.548	0.476	1.181	0.275
SD bootstrap	0.053	0.049	0.235	0.033	0.063	0.067	0.161	0.029
Var( $\hat{\omega }$)	6.612	2.066	6.005	0.993	5.078	1.210	3.849	1.399
Mean bootstrap	7.296	2.003	6.581	1.005	5.439	1.209	3.683	1.319
SD bootstrap	2.111	0.473	1.376	0.173	1.120	0.229	0.568	0.258

## 7 Conclusion

The methods in this paper are applicable for analysis of trials with selective crossover arising due to results from early efficacy endpoints and preserve randomisation. Our binary estimator is relatively simple to implement, but the proportional‐hazards model is more involved. An R package 13 with code for both is available on request. We end by discussing the model assumptions in relation to other methods, and comment on other breast cancer trials where treatment efficacy might be estimated.

The most common methods to account for selective crossover in practice are IPCW 14 and g‐estimation 15, 16. IPCW censors individuals when they cease to comply with randomised allocation, but up‐weights those who do comply and have similar characteristics to the non‐compliers, as judged by a chosen statistical model. The problem is that the assumptions required to guarantee that randomisation is maintained cannot be verified because it is not possible to check the randomised balance of factors that have not been measured. G‐estimation involves predicting the outcome for each person who switches treatment to be that expected if they had not switched. It is often accomplished through an accelerated failure‐time model 17. In contrast with IPCW, randomisation is maintained. A disadvantage is that it impossible to assess the assumed effect of treatment on the insistors after the offer to switch because there is no comparison group: all insistors receive treatment after the offer. It is also not possible to allow for time‐dependent treatment effects. Other advantages and disadvantages of these methods have been discussed more fully elsewhere 18, 19.

Although inverse probability weighting and g‐estimation make important untestable assumptions that are not required by our method, they may have better statistical performance than our estimators when their assumptions are most closely met. For instance, assumptions A5b and A6 might be restrictive for a particular data set. We also note that assumption A1 precludes a stochastic time of crossover, perhaps arising due to the health of a participant though time when randomised to control, but inverse probability weighting and g‐estimation do not require this assumption. An important example of this in oncology is crossover after progression‐free survival in advanced disease.

Other oncology trials appear to be suitable for our estimators. The open‐label ABCSG‐8 addressed a similar issue to BIG‐1 98 and after publication of initial results approximately 9% crossed over from the tamoxifen arm 20. The MA.17 trial showed superiority of letrozole over placebo for postmenopausal women with hormone receptor‐positive breast cancer following about 5 years of tamoxifen 21 and almost two‐thirds of the patients in the control arm crossed over after unblinding. In the open‐label Herceptin Adjuvant (HERA) trial, more than half of patients in the ‘control’ group chose to cross over after a first interim analysis 22. With such substantial crossover, methods such as those in this article are clearly needed to help assess longer‐term differences between treatments.

In conclusion, we recommend that treatment efficacy and our estimators thereof be considered for trials with selective crossover in one arm arising due to results from early efficacy endpoints.
